# CARING FOR NURSING HOME RESIDENTS WITH DEMENTIA AND SERIOUS MENTAL ILLNESS: A QUALITATIVE STUDY

**DOI:** 10.1093/geroni/igae098.3824

**Published:** 2024-12-31

**Authors:** Laura Block, Andrea Gilmore-Bykovskyi, Donovan Maust, Tonya Roberts

**Affiliations:** University of Wisconsin-Madison, Madison, Wisconsin, United States; University of Wisconsin-Madison School of Medicine and Public Health, Madison, Wisconsin, United States; University of Michigan, Ann Arbor, Michigan, United States; University of Wisconsin - Madison, Madison, Wisconsin, United States

## Abstract

Over half of nursing home (NH) residents are living with dementia, and a growing number also experience serious mental illness (SMI) and/or comorbid dementia and SMI. Despite the heightened vulnerability of this population, studies indicate NHs struggle to meet their needs, yet little research has documented specific challenges or strategies for their care. By employing a qualitative descriptive design, this study aims to describe NH staff perspectives on caring for residents with dementia and SMI. Partnerships were developed with NH and provider networks servicing rural and urban areas of a Midwest state, with purposive sampling of not-for-profit, county-run and for-profit facilities. Staff completed semi-structured interviews, which were analyzed applying thematic analysis. Ten individuals from three facilities have participated. While all participants identified dementia as common among residents, recognition of SMI as prevalent varied by staff and facility type. Participants described an interaction whereby a resident’s past (e.g., trauma) interacts with their mental health, functional, behavioral, and cognitive needs, which then influences selection of care and symptom management strategies. Staff described person-centered care as a universal best practice, while prescribing clinicians were attuned to appropriate pharmacology that reflected the resident diagnosis. Staff in some facilities described developing tailored behavioral and crisis plans. Findings suggest potential best practices to support residents with dementia and SMI, yet ongoing challenges caring for individuals with more complex behaviors and/or comorbid SMI. Future research should integrate further frontline nurse and nursing assistant perspectives, as additional strategies and challenges translating care plans to practice may exist.

